# Detection of NP, N3 and N7 antibodies to avian influenza virus by indirect ELISA using yeast-expressed antigens

**DOI:** 10.1186/1743-422X-6-158

**Published:** 2009-10-07

**Authors:** Chitra Upadhyay, Arun Ammayappan, Vikram N Vakharia

**Affiliations:** 1 Center of Marine Biotechnology, University of Maryland Biotechnology Institute, 701 East Pratt Street, Baltimore, Maryland 21202-3101, USA; 2 Department of Veterinary Medicine, University of Maryland, College Park, Maryland 20742, USA

## Abstract

**Background:**

Avian influenza viruses, belonging to the family Orthomyxoviridae, possess distinct combinations of hemagglutinin (H) and the neuraminidase (N) surface glycoproteins. Typing of both H and N antigens is essential for the epidemiological and surveillance studies. Therefore, it is important to find a rapid, sensitive, and specific method for their assay, and ELISA can be useful for this purpose, by using recombinant proteins.

**Results:**

The nucleoprotein (NP) and truncated neuraminidase subtype 3 and 7 of avian influenza virus (AIV) were expressed in *Saccharomyces cerevisiae *and used to develop an indirect enzyme-linked immunosorbent assay for antibody detection. The developed assays were evaluated with a panel of 64 chicken serum samples. The performance of NP-ELISA was compared with the commercially available ProFlok^® ^AIV ELISA kit. The results showed comparable agreement and sensitivity between the two tests, indicating that NP-ELISA assay can be used for screening the influenza type A antibody in AIV infected birds. The N3 and N7- ELISAs also reacted specifically to their type specific sera and did not exhibit any cross-reaction with heterologous neuraminidase subtype specific sera.

**Conclusion:**

The study demonstrates the expression of the NP, N3, and N7 proteins of AIV in yeast (*S. cerevisiae*) and their application in developing an indirect ELISA for detecting NP, N3 and N7 antibodies from AIV-infected chicken sera. The described indirect ELISAs are rapid, sensitive, specific and can be used as promising tests during serological surveillance.

## Background

Influenza viruses belong to the *Orthomyxoviridae *family. The influenza viruses are characterized into three types: A, B and C, based on the structural (nucleoprotein [NP]) and matrix [M] proteins ([1]. Type A viruses are further classified into subtypes based on the surface glycoproteins; hemagglutinin (HA or H) and neuraminidase (NA or N) [2]. Currently, sixteen HA subtypes (H1-H16) and nine NA subtypes (N1-N9) have been recognized [3-7]. The virus has been recovered from domestic and wild avian species throughout the world and has high impact on international trade in poultry and poultry products [2,8,9].

From late 2003 to July 2009, AI outbreaks in poultry has been reported in Asia, including China, Vietnam, Thailand, India, Bangladesh and other countries ([10,11]. AIV has become an important potential risk factor for human health. Since May 2005, the numbers of both affected countries and confirmed cases of influenza A (H5N1) virus infection in humans have been increased [12].

Serological surveillance of antibodies against AIV is of great importance in preventing and controlling AI. Identification of both H and N subtypes is highly essential for the epidemiological and surveillance studies [13]. The widely used agar gel immunoprecipitation (AGP) test detects antibodies to both the NP and M proteins. However, the test has lower sensitivity as compared to the ELISA and HI tests [14] and it requires large quantities of both antigen and antibody to form the precipitation line. Hemagglutination inhibition (HI) and neuraminidase inhibition (NI) assays are the commonly used tests for detection of H or N subtypes and both these tests are laborious and time consuming. In addition, there are no standard reagents available for these tests, which lead to lab to lab variation in the interpretation of the result, thus demanding alternative tests which should be more accurate, reliable and replicable. HI test is also limited to detect hemagglutinin subtype and requires working with live virus, which is of major concern as it may allow dissemination of thevirus. The tests currently available for detection of AIV, such as the Flu Detect (Synbiotics) FLU OIA TEST (Biostar) and the Directigen FLU A kit (BD, Biosciences) [15,16], are based on the detection of the viral nucleoprotein, which is conserved in all influenza A viruses and does not provide the information about the circulating neuraminidase subtypes. Thus, it is important to develop a serological assay which is safe, sensitive, specific, cost-effective, and easy to perform, especially for its application in developing and underdeveloped countries.

Recombinant proteins of AIV have been produced in expression systems using prokaryotic or eukaryotic host organisms [17-21]. The commonly preferred models for protein expression are the *E. coli *and insect-cell culture systems. Yeasts expression systems are attractive alternatives for the production of heterologous proteins. Their eukaryotic subcellular organization allows them to carry out the post-translational folding, processing and modifications required to produce bioactive mammalian proteins. In addition, yeast combines the simplicity and cost-effectiveness of bacterial expression systems and is better than the more expensive and less convenient cell culture systems [22]. To date, there are no reports of using yeast expressed AIV recombinant proteins as antigens for ELISA. To the best of our knowledge, this is the first report of expressing NP, N3 and N7 proteins of AIV in *S. cerevisiae *and the development of an indirect ELISA test.

## Methods

### Serum samples

A panel of sera containing 64 chicken serum samples, of which 24 were AIV-positive and remaining 40 were AIV-negative, was used in this study. AIV positive sera were purchased from National Veterinary Service Laboratory, Iowa USA (Table 1). Positive sera for Newcastle disease (ND), infectious bursal disease (IBD) were obtained from Synbiotics Inc., CA USA and serum for infectious bronchitis (IB) was purchased from Charles River Laboratories, MA USA. Out of 40 serum samples, 37 were from chickens negative for AIV (Georgia Poultry Laboratory, GA USA, and Synbiotics, Inc., CA USA) and the rest were positive for Newcastle disease (ND), infectious bursal disease (IBD) and infectious bronchitis (IB) obtained from Synbiotics and Charles River Laboratories respectively.

**Table 1:** List of strain or source used for raising AIV serum employed in this study.

**Type**	**Strain or Source**	**Type**	**Strain or Source**
N1	A/EQUINE-1-SW/TENN/3/76 (H7N1)	H4	A/Mynah/Mass/71 (H4N8)

N2	A/TY/Mass/65 (H6N2)	H5	A/TY/Wisconsin/68 (H5N9)

N3	NWS-NAV2/Nws-TY/England (H0N3)	H6	A/TY/Ontario/63 (H6N8)

N4	A/TY/Ontario/6118/67 (H8N4)	H7	A/TY/Oregon/71/(H7N3)

N5	NWS-N5 (H0N5)	H8	A/TY/Ontario/6118/67 (H8N4)

N6	NWS-DK/ENG/56 (H0N6)	H9	A/Gull/MD/4435/80 (H9N5)

N7	A/CK/Germany "N"/49 (H10N7)	H10	A/CK/Germany/"N"/49 (H10N7)

N8	Nws-Eq-2 (H0N8)	H11	A/DK/Memph/546/74 (H11N9)

N9	A/DK/Memph/546/74 (H11N9)	H12	A/DK/Alberta/60/76 (H12N5)

H1	NJ/8/76-EQ (H1N7)	H13	A/Gull/MD/704/77 (H13N6)

H2	A/Pintail/Alberta/293/77 (H2N9)	H14	A/Mallard/Gurjev/263/82-A/BEL/42 (H14N1)

H3	A/DK/Ukraine/1/63 (H3N8)	H15	A/Shearwater/W.Australia/2576/79 (H15N9)

### Cloning of AIV genes

The NP gene [A/Ck/HK/CSW161/2003 (H9N2)] was amplified by reverse transcription-polymerase chain reaction (RT-PCR) using the upstream primer NPEcoF (5'-CGAATTCATGGCGTCTCAAGGCACCAAACGA-3') and downstream primer NPHisR5'-CAAGCTTCAATGGTGATGGTGATGATGATTGTCATACTCCT-3'). Both the primers contained EcoRI site at their 5' and 3' end, respectively and a 6× Histidine tag was incorporated at the C-terminus of the NP gene. Similarly, the truncated N3 [A/mallard/duck/ALB/279/1977 (H7N3) and N7 genes [A/pintail/duck/ALB/303/1977 (H10N7)] were amplified using their respective primer pairs (N3TBamF:5'-GGGATCCATGACAGATGGCCCTGCTGCTAATAG-3'; N3TXhoR:5'-CCTCGAGGCCCTTGGTTTTGACAATGAAAC-3' and N7TBamF:5'-GGGATCCATGACCGACGGATCGGCTAGTAG-3'; N7TXhoR:5'-CCTCGAGTTCACTGTTATCATTCCAATAGTC-3').

The amplified products were cloned into the pCR2.1 vector (Invitrogen, CA, USA) following manufacturer's instructions and called as pCR-NP, pCR-N3/N7 plasmids. The integrity of the plasmid was verified by sequencing the DNA using an automated DNA sequencer (Applied Biosystems, CA, USA). The pCR-NP plasmid was digested with EcoRI and the NP gene was cloned in the unique EcoRI site of *S. cerevisiae *expression vector pESC-URA (Stratagene, La Jolla, CA) downstream of the GAL10 promoter. The amplified truncated neuraminidase subtype 3 (N3T) and 7 (N7T) genes were subcloned between BamHI and SalI sites of pESC-URA vector downstream of the GAL1 promoter. The inserts (~0.525 kb fragment) were ligated in-frame with the c-Myc tag (present in the vector) at the C terminus of N3T and N7T proteins.

### Expression of NP, N3, and N7 proteins in yeast

The pESC-derived expression plasmids carrying the NP, N3 and N7 protein-encoding sequences were transformed into the *S. cerevisiae *(strain MYA378; Stratagene, La Jolla, CA) using the Yeast Transformation Kit (Sigma, St Louis, MO) following the manufacturer's instructions. Transformed yeast colonies were selected for growth in synthetic complete medium without uracil and expression of the genes was induced with 2% galactose. Expression of proteins was checked by SDS-PAGE and western blot assays. Briefly, the yeast colonies were grown at 30°C for three days collected by centrifugation, and resuspended in 100 μl 1× loading buffer and boiled for 5 minutes. After centrifugation, the supernatant was analyzed by 12% SDS-polyacrylamide gels. The gel was transferred to nitrocellulose (NC) membrane by electroblotting. The NC membrane was blocked with 5% skim milk (SM) and incubated at 37°C for 1 hour with AIV polyclonal or monoclonal (polyhistidine or c-Myc) antibody (Sigma, St. Louis, MO). After washing three times with PBST buffer (PBS pH 7.4 with 0.1% Tween-20), the NC membrane was incubated with alkaline phosphatase-labeled secondary antibody (KPL, Gaithersburg, MD). Detection was obtained using the colorimetric substrate Fast red and Napthol in 0.1 M Tris-HCl buffer pH 8.0.

### Isolation of recombinant NP, N3, and N7 proteins from yeast

The *S. cerevisiae *culture expressing NP protein was expanded in uracil deficient drop-out media containing 2% galactose and was grown to an OD600 of 0.8. Large scale cultures of 20 L were collected and the pellet was cracked with microfluidizer. The clarified supernatant was used for coating the ELISA plates. For N3 and N7, 2 L of yeast cultures were centrifuged at 3,000 × *g*, and the pellet (~10 gm) was resuspended in 450 ml of 0.2 N NaOH solution, and incubated for 5 min at room temperature. The suspension was then centrifuged at 2,500 × *g *for 5 min and the pellet was treated with 12.5 ml lysis buffer [50 mM Tris-Cl (pH 8.0), 150 mM NaCl, 0.02% sodium azide, 1% NP-40, 100 μg/ml PMSF and 1 μg/ml aprotinin] for 30 min at 4°C, followed by centrifugation at 9000 × *g *for 20 min. The supernatant was collected and stored at -80°C until use. The N3 and N7 proteins were purified using c-Myc tagged affinity purification columns (MBL, Japan) and the purified protein was used for coating the ELISA plates.

### Enzyme-linked immunosorbent assay (ELISA)

AIV positive chicken antisera purchased from Charles River Laboratories and negative sera obtained from Synbiotics Inc. was used to optimize the NP-ELISA. The crude NP, purified N3, and N7 proteins were coated onto separate 96-well plates in PBS (pH 8.5) overnight at 4°C. The optimal antigen concentration and the optimal serum dilution were determined by checkerboard titration of each antigen (1:25, 1:50, 1:100, 1:200, 1:400,1:800, 1:1600) and antisera (1:25, 1:50, 1:100, 1:200, 1:400,1:800). The wells were washed three times with 0.05% Tween-20-phosphate-buffered saline (PBST) and subsequently blocked with PBST supplemented with 3% (w/v) skimmed milk powder (SMP) for 10 minutes at room temperature (RT). The positive and negative sera were added, followed by incubation for 30 min at RT. Subsequently, the plates were washed three times with PBST, followed by the addition of goat anti-chicken IgG HRP secondary antibody diluted 1:500 (KPL, Gaithersburg, MD) in PBST with 0.05% gelatin. After 30 min incubation at RT the plates were washed as described above, and 100 μl/well of ABTS substrate (2, 2'-azino-bis (3-ethylbenzthiazoline-6-sulfonic acid), (KPL, Gaithersburg, MD) was added. After 15 min incubation, the reaction was stopped by addition of 100 μl/well of 1% SDS. The optical density at 405 nm (OD405) was measured with an automated plate spectrophotometer (Thermo Lab Systems, Multiskan, Finland).

### The positive threshold and specificity tests

The ELISA plates were coated with optimal NP/N3/N7 antigen concentration and sera at the optimal dilution were added. The ELISA was performed as described above. The 37 negative sera were used to establish the endpoint cut-off value as the mean OD405 of the 37 negative sera plus 3 standard deviations (mean+3SD). To determine the specificity of NP/N3/N7-ELISA, a panel of sera positive for Newcastle disease, infectious bronchitis and infectious bursal disease was tested.

### Optimal Positive/negative ratio and Standard curve

The standard curve was constructed using the P/N ratio method, according to Briggs and Skeeles [23]. Briefly, the OD405 value of the positive serum (P value) was divided by the OD405 value of the pooled negative sera (N value). A regression line was constructed for each serum sample and the dilution at which P/N = 1 was determined. This dilution was called ELISA titre (ET) and was determined for each serum sample. According to Briggs et al. [24], two points were recorded from the regression lines for each serum sample, the P/N ratio at 1:100 dilution and the ELISA titre. Using these two points, the ETs were plotted against P/N ratios at 1:100 and the prediction equation was determined.

## Results

### Expression of the recombinant AIV protein

**Figure 1:**
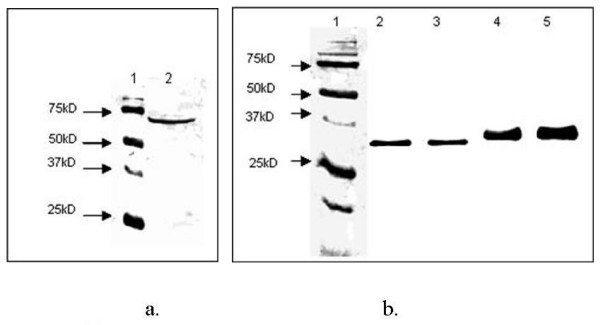
**Immunoblot analysis of yeast lysate from recombinant yeast clones**. The proteins were separated by SDS-PAGE on a 12% gel, blotted on to nitrocellulose, reacted with respective antibodies, and detected with colorimetric substrate Fast red and napthol alkaline phosphatase (Fig. 1a). Immunoblot of nucleoprotein (NP) probed with polyhistidine monoclonal antibody. Lane 1, molecular weight marker and lane 2, recombinant yeast cell lysate expressing (NP) (Fig. 1b). Immunoblot of truncated neuraminidase 3 (N3T) and neuraminidase 7 (N7T) probed with c-Myc monoclonal antibody. Lane 1, molecular weight marker; lane 2, recombinant yeast expressing N3T; lane 3, affinity purified N3T; lane 4, recombinant yeast expressing N7T; lane 5, affinity purified N7T. The position of the marker proteins in kD (right) are indicated.

The expression of the NP, N3 and N7 genes was induced by 2% galactose and the yeast samples were collected after 72 hours. Subsequently, the yeast lysates were analyzed by Western blot analysis. The NP protein reacted specifically at approximately 64 kD position when probed with polyhistidine MAb (Fig 1a). The N3 and N7 proteins gave specific bands at ~32-35 kD position with c-Myc MAb (Fig 1b). The blots were also probed with AIV polyclonal antibody and the proteins reacted at the expected position (NP at 64 kD and N3/N7 at 32-35 kD position; data not shown). The N3 and N7 proteins were purified using c-Myc affinity purification gel. The protein concentration of NP and purified recombinant N3T/N7T was measured and the samples were stored at -80°C until use.

### Optimization of ELISA Procedure

**Figure 2:**
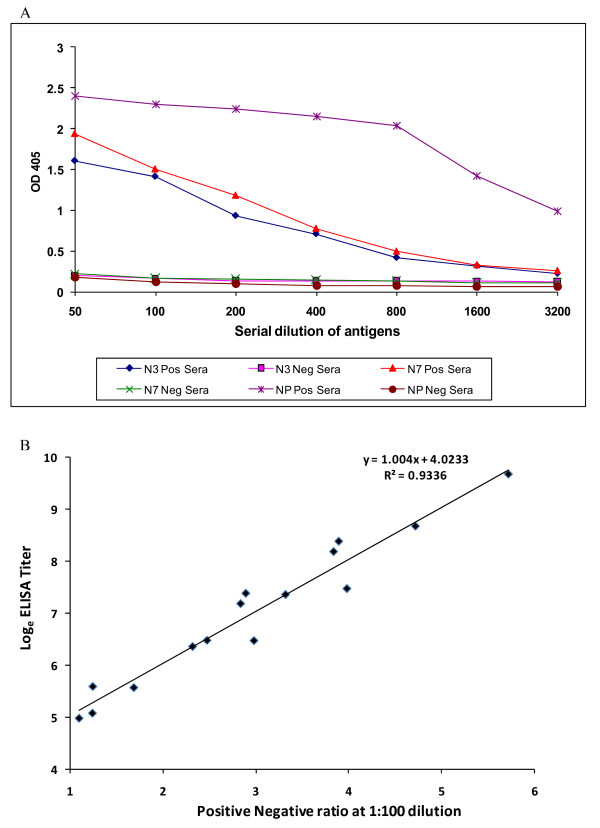
**a. Serial dilution of antigens from 1:50 to 1:3200**. All antibodies are at dilution 1:100. Selected Ag concentration for NP (1:400 dilution), N3 and N7 (1:100 dilution) antigen for cross-reactivity study are 80 μg/ml, 60 μg/ml and 50 μg/ml, respectively. **b. Linear regression of ELISA titre and positive/negative ratio (P/N) ratio of 1:100 serum dilution. **The regression equation y = 1.004x + 4.0233 (r = 0.936) is shown, where y is the ELISA titre of an individual serum sample, and x is the P/N value at 1:100 serum dilution.

To determine the optimal concentration of the antigen and the test serum, checkerboard titration was performed. The recombinant proteins were immobilized on ELISA plates in two-fold serial dilution ranging from 1:50 to 1:3200 and the antisera was also diluted in serial two-fold dilutions ranging from 1:25 to 1: 3200. The optimal antigen concentration of NP, N3 and N7, as determined by checkerboard titration, was 80 μg/ml (1:400), 60 μg/ml (1:100) and 50 μg/ml (1:100), respectively (Fig 2a). The optimal serum dilution was obtained by serial titration of high positive serum (OD ≥ 1) and the pooled negative serum from dilution 1:25 to 1:12800. The OD405 was plotted against the dilutions and the dilution 1:100 was selected as the dilution for determining the P/N ratio as at this dilution the OD of the negative serum was very low, whereas the positive serum had a high OD405 reading. To test the optimal coating buffer, 0.05 M bicarbonate/carbonate buffer (pH 9.6), PBS (pH 7.4) or PBS (pH 8.5) was screened. It was found that PBS (pH8.5) was the best coating buffer for immobilization of the proteins.

### The ELISA positive threshold

The end-point cut-off was established by titration as the mean OD405 value of the 37 negative sera plus 3 standard deviations (mean + 3SD). The OD405 values of sera ranged from 0.135 to 0.190. With NP antigen, the mean OD405 value and SD were 0.163 and 0.031, respectively. Thus, the positive threshold determined for NP-ELISA was 0.256. A serum sample was considered positive when its OD405 value at optimal dilution (1:100) was greater than the positive threshold. Similarly, for N3 and N7-ELISA the end-point cut-off value determined was 0.341 and 0.413, respectively.

### Specificity tests

**Figure 3:**
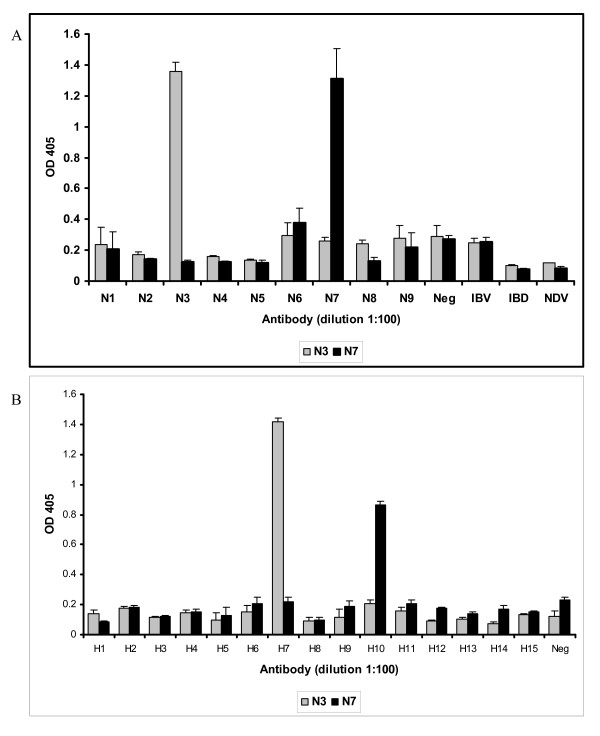
**Reactivity of N3T and N7T antigens with (a) neuraminidase-positive AIV serum and (b) hemagglutinin-positive AIV serum**. The ELISA plates were coated with N3T and N7T antigens at 60 μg/ml and 50 μg/ml, respectively. The coated plates were reacted with serum samples at single 1:100 dilution and incubated for 30 min at room temperature, washed three times with PBST (PBS with 0.05% Tween-20), followed by 30 min incubation with goat anti-chicken IgG HRP-conjugated secondary antibody. The reaction was developed with ABTS (2, 2'-Azinobis [3-thylbenzothiazoline-6-sulfonic acid]-diammonium salt) substrate, stopped after 15 minutes with 1% SDS, and the plates were read in a spectrophotometer using the filter at 405 nm.

The specificity of NP-ELISA was evaluated by testing with panel of sera containing antibodies against AIV, NDV, IBV and IBDV. The results show that the NP-ELISA reacted specifically with all the 24 AIV-positive sera and there was no cross-reaction with antisera against NDV, IBDV and IBV. The specificity of the N3 and N7 antigens was tested using the AIV sera, which are positive for all nine N subtypes and 15 H subtypes (Table 1). The antigens reacted specifically to their respective serum and did not cross-react with any heterologous sera. Both the antigens did not react with the antisera against IBDV, IBV and NDV (Fig. 3a and 3b). This result demonstrates that the antigens have high specificity.

### Standard curve

The regression line of each serum was constructed using the P/N ratio (data not shown). The ET of each serum was calculated from this analysis and a standard curve [y (ET) = 1.004x (P/N value at 1:100 serum dilution) + 4.0233, R^2 ^= 0.9336 was developed (Fig. 2b). The correlation coefficient (*r *value) between ET and P/N at 1:100 reached at 0.9336, indicating that single serum dilution can be used to derive the ELISA titers of antibodies.

### Comparisons of NP-ELISA with ProFlok PLUS test

The NP-ELISA was compared with the commercially available ProFlok PLUS test kit (Synbiotics Inc., CA USA), the results revealed comparable sensitivities, specificities and overall agreements (Table 2). The NP-ELISA showed 98% agreement and 97% specificity with the ProFlok PLUS test.

**Table 2:** Comparative evaluation of NP-ELISA with ProFlok PLUS ELISA kit for detection of anti-AIV antibodies.

**Test**	***N*^*a*^**	**% agreement^*b*^**	**% sensitivity^*c*^**	**% specificity^*d*^**
NP-ELISA	64	98 (63/64)	100 (24/24)	97 (40/41)

^*a *^Number of antisera tested.

^*b *^(no. of samples positive by both method + no. of samples negative by both methods)/total no. samples × 100.

^*c *^True positive/(true positive + false negative) × 100.

^*d *^True negative/(true negative + false positive) × 100

## Discussion

The accurate and prompt diagnosis of AIV infection in birds is a critical component of surveillance and disease control strategy. Currently, virus isolation in embryonated eggs or in cell culture followed by subtyping are considered as the standard protocol for detecting avian influenza virus. However, the conventional culture method requires special collection and transport conditions to maintain the viability of the virus and the results may take 1 to 2 weeks, by that time the results may not be relevant. Molecular methods, such as reverse transcription polymerase chain reaction [25-29] real-time RT-PCR [30-34], RT-PCR enzyme-linked immunosorbent assay (RT-PCR-ELISA) [5,35-37], have also been used for AIV diagnosis. Real-time PCR assays and a DNA microarray analysis for the detection of influenza virus have also been developed [38] but it is useful only as a supplement to PCR-based diagnostic methods. Although, these methods are rapid, sensitive, and specific but the test results often need to be confirmed by additional tests, such as sequence analysis of the PCR-amplified products.

Additionally, a recent report documents the failure of real-time RT-PCR protocol [39], currently used in national avian influenza surveillance programs, to detect subtype H7 avian influenza viruses isolated from wild birds. The findings depict the unreliability of the test for surveillance programs and elucidate the need for new reliable diagnostic testing methods. Antigen capture immunoassay has been another commonly used tool for the diagnosis of AIV [40,41]. The antigen capture tests available are intended for testing AIV infection in humans but are also being used for veterinary purposes. Almost all the tests target the well conserved nucleoprotein and do not provide the information about the circulating neuraminidase subtypes. Surveillance for avian influenza becomes much more difficult because of the inability to differentiate the infected from vaccinated animals (DIVA). The DIVA strategy, although originally proposed in 1986 [42], has only been recently put into practice. Among the different DIVA strategies, one approach is to use an inactivated oil emulsion vaccine containing the same hemagglutinin (H) subtype as the field virus, but a heterologous NA subtype. Serologic surveillance can then be performed for the homologous NA subtype as evidence of natural infection. This novel strategy was first used in Italy in 2000 during an H7N1 outbreak where the turkeys were primarily vaccinated with an H7N3 vaccine. The only field validated DIVA test, based on indirect immunofluorescence assay [43,44], has limitations to be set up as high throughput screening test and the assay requires subjective interpretation of the results. Thus, it is important to develop a serological assay for DIVA, which is sensitive, specific, cost-effective, and easy to perform, especially for its application in developing and underdeveloped countries.

In the present study, attempts were made to develop ELISA for detecting antibodies against N3, N7 and NP protein of AI viruses. Neuraminidase proteins are insoluble and very difficult to express. Moreover, they share high percentage similarity which makes it difficult to utilize these proteins in ELISA system. We successfully expressed truncated N3 and N7 proteins in *S.cerevisiae *in a way to minimize the cross-reaction between the other neuraminidase types. Further, we used these proteins to develop indirect ELISA for detecting antibodies against N3 and N7. The specificity of the N3 and N7 antigens was tested using the AIV sera, positive for all nine N types and 15 H types. The antigens reacted specifically to their respective serum and did not cross-react with any heterologous sera or exhibited negative reactivity with antisera against IBDV, IBV and NDV. The high specificity of N3 and N7 ELISA demonstrates their suitability to be used for differentiating infected and vaccinated birds.

The other ELISA we developed in this study is based on nucleoprotein (NP), which is highly conserved among AI and can be used as candidate diagnostic antigen because of its group and type specificity. There are previous reports of NP ELISA [17,18,45,46], which use either monoclonal antibodies or the recombinant proteins that are expressed in *E.coli *and baculovirus. In this study, we exploited the advantages of yeast expression system. The full-length NP protein expressed in *S. cerevisiae *was used to develop an indirect ELISA assay to detect AIV type-specific antibody. The ELISA developed in this study is cost-effective and can be performed at a single serum dilution, thus reducing the error inherent to serial dilutions. The result of comparative evaluation among NP-ELISA and ProFlok PLUS ELISA showed 98% agreement and 97% specificity, proving that the NP-ELISA system developed in this study is as effective as the commercially available one.

In the present study, we utilized the P/N ratio regression lines to derive the predication equation from which the NP-ELISA titre (ET) of a serum sample could be determined using one dilution. The co-relation coefficient (*r *value) between ET and P/N at 1:100 reached at 0.9336, indicating that the ELISA titres of antibodies could be derived at one serum dilution. The NP-ELISA can rapidly evaluate levels of antibodies to AIV and is suitable to detect large numbers of serum samples that would provide scientifically relevant information during a surveillance program to control AIV infections in poultry.

To further strengthen this study, the ELISA system developed based on yeast-expressed antigens was compared with another ELISA system based on baculovirus-expressed antigens (NP, N3, and N7). The recombinant protein expressed in insect cell culture system showed high level of background as compared to the yeast expressed proteins (data not shown), suggesting its unsuitability to be used in indirect ELISA system. A different approach was tried for protein extraction for N3 and N7 from yeast (0.2 N NaOH) and it did not have any negative effect on the quality of the product. This method is simple and relatively inexpensive and bypasses the necessity of the microfluidizer and glass-bead or enzymatic disruption of the yeast cell wall without compromising the quality and quantity of the extracted protein.

## Conclusion

In conclusion, this study demonstrated the expression of the NP, N3, and N7 proteins of AIV in yeast (*S. cerevisiae*) and their application in developing an indirect ELISA to detect NP, N3 and N7 antibodies from AIV-infected chicken sera. With the high degree of conservation and group-specificity of NP, the NP-ELISA developed in this study could detect AI antibodies to all H (1-15) and N (1-9) subtypes. The N3 and N7 ELISAs reacted specifically to their type-specific sera and exhibit no cross-reactivity to other neuraminidase subtypes, indicating high specificity. Furthermore, the tests can be completed within 2 hours and the ELISA system developed in this study could serve as a rapid, accurate and inexpensive diagnostic tool during surveillance and epidemic outbreaks.

## Competing interests

The authors declare that they have no competing interests.

## Authors' contributions

VNV conceived the study. CU planned the experimental design, CU and AA carried out cloning and sequencing. CU drafted the manuscript. All authors critically reviewed and approved the final manuscript.
